# Associations Between Interindividual Differences, Expectations and Placebo and Nocebo Effects in Itch

**DOI:** 10.3389/fpsyg.2021.781521

**Published:** 2021-12-13

**Authors:** Stefanie H. Meeuwis, Henriët van Middendorp, Dieuwke S. Veldhuijzen, Andrea W. M. Evers

**Affiliations:** ^1^Pain Research Group, Institute of Psychology, Jagiellonian University, Kraków, Poland; ^2^Health, Medical and Neuropsychology Unit, Institute of Psychology, Faculty of Social and Behavioural Sciences, Leiden University, Leiden, Netherlands; ^3^Leiden Institute for Brain and Cognition, Leiden University Medical Center, Leiden, Netherlands; ^4^Department of Psychiatry, Leiden University Medical Center, Leiden, Netherlands; ^5^Medical Delta Healthy Society, Leiden University, Technical University Delft, Erasmus University Rotterdam, Rotterdam, Netherlands

**Keywords:** itch, placebo effects, nocebo effects, expectations, pruritus, verbal suggestions, moderated mediation

## Abstract

**Introduction:** Placebo and nocebo effects are positive and negative health outcomes that can be elicited by the psychosocial context. They can be mediated by expectations, and may emerge in somatic symptoms even when people are aware of these effects. Interindividual differences (e.g., in personality, affective states) could impact placebo and nocebo responding, but findings are inconsistent.

**Methods:** The current work examined expectation as a mediator of the association between verbal placebo and nocebo suggestions (VSs) and histamine-induced itch across three experimental studies. Moreover, we examined whether interindividual differences (e.g., in optimism, neuroticism, behavioral activation system (BAS), body ignorance) modulated: (1) the direct association between VSs and itch (direct moderation), and (2) the indirect, expectation-mediated association between VSs and itch (moderated mediation). Positive VSs were compared to neutral instructions (Study 1; *n* = 92) or negative VSs (Studies 2+3; *n* = 203) in an open-label (i.e., explaining placebo and nocebo effects) or closed-label (concealed) context using PROCESS. First, mediation of VSs effects on itch by expectations was tested. Next, moderation by individual traits was explored using conditional process analyses.

**Results:** The effects of VSs on itch were significantly mediated by expectation in Study 1 and in the open-label (but not closed-label) contexts of Studies 2 and 3. Ignorance of bodily signals marginally moderated the direct effects of VSs on itch when closed-label suggestions were given: at low levels of body ignorance, effects of positive and negative VSs were stronger. Moreover, moderated mediation was observed in the open-label groups of Studies 2 and 3: The expectation-mediated effects of VSs on itch were stronger when BAS drive was lower.

**Conclusion:** Overall, the effects of VSs on itch were mediated by expectations in the open-label, but not the closed-label context. Moreover, the current work suggests that placebo and nocebo effects may be moderated by ignorance of bodily signals and the BAS. There was limited evidence that other interindividual differences modulated placebo and nocebo responding in itch.

## Introduction

Placebo effects are positive health outcomes such as reduced somatic symptoms (e.g., pain, itch, or nausea) that cannot be attributed to active treatment components, but are elicited by psychosocial and contextual factors that signal potential treatment benefits (Evers et al., 2018; Wolters et al., 2019; Mitsikostas et al., 2020). Nocebo effects can be described as the opposite—adverse health outcomes, for instance, increases in somatic complaints or treatment side effects, or decreased treatment efficacy, which can be elicited by psychosocial factors signaling potential drawbacks of a treatment (Mitsikostas et al., 2020). Research generally discerns three mechanisms that mediate placebo and nocebo effects: (Conscious) expectation, associative learning, and observational learning (Rossettini et al., 2020; Evers et al., 2021). Expectations about treatment outcomes can be modulated by verbal suggestions. Experimental studies, for instance, show that verbal suggestions of pain relief can influence expectations and can lead to analgesia following administration of an inert intervention (Petersen et al., 2014; Colloca and Barsky, 2020). Similarly, positive verbal suggestions can reduce symptoms of itch (Bartels et al., 2016; Wolters et al., 2019). When placebo effects are elicited by associative learning, or conditioning, an individual learns that a certain cue (e.g., the treatment context, or a medical ritual) and positive health outcome (e.g., a reduction in symptoms) are associated through experience (Colloca and Barsky, 2020), whereas in observational learning, this association is learned by observing it in others (Bajcar and Ba̧bel, 2018).

Differences are observed in the magnitude of placebo and nocebo effects that can be elicited in individuals, which may be attributed to psychosocial and contextual factors. Among others, psychological traits and affective states can contribute to placebo and nocebo responsiveness (Colagiuri et al., 2015; Hall et al., 2015; Anderson and Stebbins, 2020; Frisaldi et al., 2020). With regard to these interindividual differences, optimism appears to most consistently contribute to placebo responding in pain (Geers et al., 2005, 2007, 2010; Morton et al., 2009; Darragh et al., 2014; Corsi et al., 2016), whereas anxiety seems to play a role in eliciting nocebo effects in particular (Aslaksen and Lyby, 2015; Corsi et al., 2016; Kern et al., 2020; Thomaidou et al., 2021). The evidence for the contribution of other interindividual differences, including those in personality traits of the Big Five model (i.e., neuroticism, extraversion, openness to experience, conscientiousness, agreeableness), (disposition to) worrying, or subjective stress, is more inconsistent: some studies report significant associations and other studies refute them (see, for example, Corsi and Colloca, 2017; Locher et al., 2019; Kern et al., 2020). Potentially, interindividual differences in these traits and states could influence placebo and nocebo effects. Finally, the behavioral inhibition system (BIS) and behavioral activation system (BAS) may also play a role in placebo and nocebo responding. These two systems are reflected in patterns of emotional and behavioral responses to attractive (e.g., rewards) and repulsive (e.g., punishments) stimuli (Corr, 2004, 2013). For instance, BAS comprises the sensitivity of the response to rewards, as well as the motivation to seek out rewards, whereas BIS comprises the tendency to avoid unpleasant stimuli (Carver and White, 1994). Both BIS and BAS have been associated with pain sensitivity and pain-related function (Jensen M. P. et al., 2015; Day et al., 2019; Sánchez-Rodríguez et al., 2021; Turner et al., 2021). Moreover, a more sensitive BAS has been associated with enhanced placebo analgesia (Schweinhardt et al., 2009; Yu et al., 2014; De Pascalis and Scacchia, 2017).

The relation between interindividual differences in psychological traits and affective states, and placebo and nocebo responding has not been investigated outside the area of pain very often, but there is some evidence that they modulate nocebo responding in itch (Bartels et al., 2016; Wolters et al., 2019). To illustrate, higher levels of depressive symptoms, trait anxiety, and worrying have been associated with nocebo effects in itch (Scholz and Hermanns, 1994; Bartels et al., 2014). As of yet it is still unclear how other interindividual differences may influence placebo and nocebo effects in itch. Investigating these associations may be particularly relevant given the high prevalence and large psychosocial burden of itch (Weisshaar, 2016), and given that itch is likely very sensitive to placebo effects (van Laarhoven et al., 2015).

According to the current theories on placebo effects mechanisms, verbal suggestions can influence symptoms because they change an individual’s expectations about a treatment outcome. Such a model implies that mediation occurs (Geers et al., 2019; Bingel, 2020). However, (conscious) expectations are not always measured in studies, and if they are, it is not often assessed whether they actually mediate the association between verbal suggestions and treatment outcomes. Importantly, when investigating which factors can predict or contribute to placebo and nocebo responding, expectations are also often omitted from the tested models. Given that expectations are central to placebo and nocebo responding, this essentially renders the models for testing modulation of these effects by interindividual differences incomplete. Current common practices are to either look for direct associations between an individual’s psychological traits or affective states and the outcome within different subgroups (e.g., separately for those receiving verbal suggestions and those not receiving them), or to test whether interindividual differences moderate the effects of verbal suggestions on the outcome directly (for an overview see Kern et al., 2020). Neither of these methods takes the potentially mediating role of expectations into account. Because of this, we do not know whether the extent to which interindividual differences modulate placebo or nocebo effects is dependent on the involvement of expectations. Placebo responses are complex, and the degree to which interindividual differences may influence them could be dependent on whether expectations change as a result of an intervention; for instance, we could hypothesize that optimism enhances placebo effects because suggestions influence expectations to a higher degree when people are more optimistic, or alternatively, because the effect of outcome expectations are stronger when people are more optimistic. If this proposition holds true, it may have implications for how we look at the role of interindividual differences in placebo responding. For instance, their role could change depending on whether placebo interventions aim to alter conscious expectations: factors that enhance expectation-mediated placebo responding may be relevant for verbal suggestions and other types of expectation-based effects, but less so when placebo effects are generated through other learning mechanisms, such as associative or observational learning (i.e., when the role of conscious expectations may be more limited).

**FIGURE 1:**
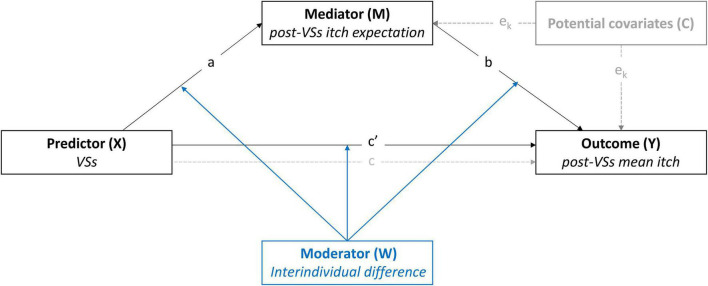
Conceptual representation of the first- and second-stage dual moderated mediation model (model 59, Hayes, 2017). The effects of positive and negative verbal suggestions (VSs) on mean itch during histamine iontophoresis were tested across the three studies. Moderation of the model by interindividual differences was tested on the indirect and direct pathways from VSs to the outcome (mean itch). The model was controlled for Pre-VSs itch expectation (studies 1–3) and baseline itch (studies 2–3). A representation of the statistical model including the tested interactions can be found in the Supplementary Material.

Investigating how interindividual differences, expectations, placebo effects and nocebo effects are interrelated could further our understanding of the manner in which interindividual differences may contribute to placebo and nocebo responding. To this end, we exploratively analyzed data of three of our previous studies that investigated placebo and nocebo effects induced by (open- or closed-label) positive and negative verbal suggestions on itch (Meeuwis et al., 2018; Meeuwis et al., 2019; Meeuwis et al., 2021). The objective was to explore the influence of interindividual differences across a mediation model of placebo and nocebo effects using conditional process analyses. Conditional process analysis can be used to test for moderation of both the direct and indirect (i.e., mediated) effects of a predictor on an outcome within a single statistical model (see Figure 1; Hayes, 2017). We hypothesized that the effects of verbal suggestions on itch would be mediated by expectations. Moreover, we expected that the strength of the associations between verbal suggestions, expectations and itch would change depending on the level of the assessed psychological traits and affective states.

## Materials and Methods

Psychological traits and affective states that may be associated with placebo and nocebo responses to verbal suggestions (VSs) in histamine-induced itch were explored across three previously published experimental studies (Meeuwis et al., 2018; Meeuwis et al., 2019; Meeuwis et al., 2021). This paper used the same data as these previous publications, but now aimed to examine interrelations between interindividual differences, expectations, and placebo and nocebo effects in itch in a larger participant sample. Supplementary Table 1 outlines the similarities and differences between the studies. Due to a large overlap in study design and VSs content, data of the second and third studies were analyzed collectively.

### Study Design

All studies’ details have been published before; short summaries of the methods are provided below.

#### Study 1. Open-Label Positive Verbal Suggestions Versus Neutral Instructions

Healthy volunteers were randomized to (1) an open-label positive VSs group or (2) a neutral instructions control group. Itch was induced experimentally during a laboratory session by 2.5 min of histamine iontophoresis. After iontophoresis, participants were asked to rate the mean amount of itch they experienced during this procedure on a 0–10 Numeric Rating Scale (NRS; 0 “no itch,” 10 “worst itch ever experienced”). Prior to iontophoresis, participants in the open-label positive VSs group were told that this procedure would elicit little itch. It was moreover explained that this suggestion of little itch may influence their experience by changing expectations about the test (open-label rationale). Participants in the control group were given neutral instructions about the study procedures instead. Before and after the VSs or neutral instructions, participants rated how much itch they expected to experience on a 0–10 NRS (as a measure of conscious expectations).

#### Studies 2 and 3. Open- and Closed-Label Positive Versus Negative Verbal Suggestions

Healthy volunteers in studies 2 and 3 were randomized to (1) an open-label positive VSs, (2) a closed-label positive VSs, (3) an open-label negative VSs, or (4) a closed-label negative VSs group. Itch was induced experimentally at baseline and following VSs by histamine iontophoresis. Mean itch was rated upon completion of this test on a 0–10 NRS (0 “no itch,” 10 “worst itch imaginable”). Participants were told that they would receive an intervention before histamine iontophoresis took place a second time (in study 2 an inert tonic was applied, and in study 3 a sham transdermal patch). Depending on group allocation, VSs of decreased (positive VSs) or increased itch (negative VSs) were given. Participants in the open-label groups additionally received an explanation of how suggestions may influence expectations: they were informed that the tonic or patch were actually sham treatments and elicit placebo effects (in case of positive VSs) or nocebo effects (in case of negative VSs). Before baseline iontophoresis and after VSs were given, participants were also asked to rate how much itch they expected to experience on a 0–10 NRS.

### Interindividual Differences

The following psychological traits and affective states were assessed across all studies: neuroticism and extraversion (Eysenck Personality Questionnaire—Revised Short Scales, EPQ-RSS; Eysenck and Eysenck, 1975), optimism (Life Orientation Test—Revised, LOT-R; Scheier et al., 1994), and the BIS and BAS subfactors drive, fun seeking and reward responsiveness (BIS/BAS scales; Carver and White, 1994). Other interindividual differences that were assessed in study 1 were subjective stress (the Perceived Stress Scale, PSS; Cohen et al., 1983), (disposition to) worrying (Penn State Worry Questionnaire, PSWQ; Meyer et al., 1990), and distress (Hospital Anxiety and Depression Scale, HADS; Zigmond and Snaith, 1983). The lie/social desirability subscale (EPQ-RSS; Eysenck and Eysenck, 1975) was additionally assessed in studies 2 and 3. Finally, attention to, and ignorance and awareness of, bodily signals (Body Attention, Ignorance and Awareness Scale, BAIAS; van Beugen et al., 2015) was measured in study 3 exclusively.

The *EPQ-RSS* subscale “neuroticism” assesses a broad personality construct that comprises emotional instability and reactivity, as well as a tendency toward anxiety and worrying (Eysenck and Eysenck, 1975; Sanderman et al., 1991). Items of this subscale include, for instance “*Does your mood often go up and down*?” The subscale “extraversion” measures a person’s tendency to be, for instance, outgoing and impulsive (example item “*Are you rather lively*?”), whereas the “lie” scale reflects a person’s tendency toward socially desirable responses (e.g., “*If you say you will do something, do you always keep your promise no matter how inconvenient it might be*?”) (Eysenck and Eysenck, 1975; Sanderman et al., 1991). Scores on these EPQ-RSS subscales range between 0 and 12, with higher scores on the neuroticism scale indicating more emotional instability and reactivity, and higher scores for extraversion indicating that the person is more extravert. Higher scores on the “lie” scale indicate that the person has a stronger tendency to provide socially desirable responses.

The *LOT-R* assesses the personality dimension “dispositional optimism,” that is, the general tendency toward expecting good outcomes (example item “*In uncertain times, I usually expect the best*”) (Scheier et al., 1994). The total score on this scale reflects a dimension from pessimism to optimism, and scores range from 0 to 24, with higher scores indicating optimism, and lower scores indicating pessimism.

The *BIS/BAS scales* comprises several subscales reflecting approach tendencies, a higher sensitivity to rewarding stimuli, and higher positive affect. “BAS drive” measures an individual’s drive or motivation in pursuing their goals (e.g., “*When I want something, I usually go all-out to get it*”), “BAS reward responsiveness” measures the sensitivity to rewarding stimuli (e.g., “*When good things happen to me, it affects me strongly*”), and “BAS fun seeking” measures the tendency and motivation to pursue pleasant or rewarding stimuli (“*I will often do things for no other reason than that they might be fun*”). The BIS subscale relates to passive avoidance, a more cautious approach to negative stimuli, and increased negative affect—particularly anxiety. Example items include “*If I think something unpleasant is going to happen I usually get pretty “worked up”* (Carver and White, 1994; Merchán-Clavellino et al., 2019; Vecchione et al., 2021). Total scores for BAS drive and fun seeking range 4–16, for BAS reward responsiveness 5–20, and for BIS 7–32. Higher scores on the BAS-trait scales reflect higher approach tendencies, and higher scores for BIS indicate a stronger tendency for passive avoidance.

The *PSS* scale assesses stress experienced within the last month (e.g., “*In the last month, how often have you been upset because of something that happened unexpectedly*?”; total score ranges 0–40) (Cohen et al., 1983); the *PSWQ* reflects an individual’s disposition to worrying (e.g., “*I worry all the time*”; total score ranges 16–80) (Meyer et al., 1990); and the *HADS* assesses depressive symptoms (“*I still enjoy the things I used to enjoy*”) and anxiety (“*I feel tense or “wound up”*”) within the past week (Zigmond and Snaith, 1983). Items are summed for the total scale “distress,” which ranges from 0 to 42. Higher scores indicate more distress.

Finally, the *BAIAS* assesses body awareness using three subscales: “body attention” (e.g., “*In general I pay attention to my physical sensations”*), “body ignorance” (e.g., “*When I am not feeling well physically, I do not know the reason”*) and “body awareness” (e.g., “*I notice changes in my body, such as whether my breathing slows down or speeds up”*) (van Beugen et al., 2015). Total scores for each BAIAS subscale are calculated by summing and then dividing for the number of subscale items, resulting in a total score between 0 and 4. Higher scores on the BAIAS subscales “body attention,” “body ignorance” and “body awareness” reflect a stronger tendency to pay attention to, to ignore, or to be aware of bodily signals, respectively.

### Statistical Analysis

Analyses were conducted using IBM SPSS version 26.0 (Chicago, IL, United States) and the syntax-driven PROCESS 3.5 SPSS macro for mediation and conditional process analyses (Hayes, 2017). All analyses were conducted separately for study 1, and combined for studies 2 and 3. Between-group differences in baseline expectations and itch, as well as in psychological traits and affective states, were checked using chi-square tests and analyses of variance (ANOVAs). Prior to the mediation and conditional process analyses, assumptions for ordinary least squares (OLS) regression analysis were checked, including linearity, homoscedasticity, independence and multivariate normality, and absence of multicollinearity.

First, direct and indirect effects of VSs on mean itch were explored in a simple mediation model with post-VSs itch expectation as mediator variable (PROCESS model 4). Next, conditional process analyses were used to explore first- and second-stage dual moderated mediation effects as well as moderation of the direct effects of VSs on itch by individual traits (PROCESS model 59; Hayes, 2017). Conditional direct and indirect effects of VSs on itch were always probed at low (16th), medium (50th), and high (84th) percentiles of the moderator. When relevant (i.e., when *p*<0.10 for moderator × group or moderator × mediator interaction), the conditional effects of VSs on itch expectation and the conditional effects of itch expectation on itch were probed for these percentiles as well. Bootstrapped 95% percentile confidence intervals (CI) were computed with a rate of 10.000 samples to assert significance of these calculated conditional effects. To ascertain whether moderated mediation was present, an index for moderated mediation was calculated for dichotomous variables (e.g., for sex). Significance of this index was then checked using the 95% bootstrap CI. Because the model we tested has multiple points where it can be moderated (see Figure 1 and Supplementary Figure 1), the function of the effects of continuous moderators on the indirect path (X→M→Y) is non-linear. This prevents computation of a single index value for moderated mediation (Muller et al., 2005; Hayes, 2015). Instead, pairwise contrasts between the indirect effects of VSs on itch were calculated at low, medium and high levels of the moderator variable. The 95% CI for these contrasts were then used to ascertain moderated mediation.

A moderation effect was deemed present when there was (1) a significant (*p*< 0.05) or marginal (*p* < 0.10) interaction in the OLS regression analysis, and (2) at least one of the effects probed at low, medium and high levels of the moderator was significant as indicated by the 95% bootstrap CI. When the standard probing of effects reveals significant effects of VSs on itch at any of the levels of the moderator, but the OLS regression did not show marginal or significant interaction effects, no moderation was present. Finally, in all mediation and conditional process models, pre-VSs itch expectation was included as a covariate on the mediator (post-VSs itch expectation) level. In addition, mean itch during baseline iontophoresis was included as a covariate in the models of studies 2–3 on the mediator level as well as on the outcome (post-VSs mean itch) level. All analyses were conducted two-sided with α < 0.05.

## Results

### Participants and Baseline Differences Between Groups

Data of 295 participants were analyzed (study 1: *n* = 92, 81.5% female, M_age_ ± *SD* = 21.3 ± 1.94; studies 2 and 3: *n* = 203, 83.3% female, M_age_ ± *SD* = 21.9 ± 2.70). No between-group differences in psychological traits, affective states, or baseline ratings of expected itch and mean itch experienced during iontophoresis were observed for study 1 (all *p* ≥ 0.11; Supplementary Table 2). Some incidental group differences were observed in the open-label arm of studies 2 and 3, which will be taken into account during the interpretation of the findings: neuroticism and BAS drive were higher in the positive compared to the negative VSs group; [*t*(98) = –2.05, *p* = 0.043; and *t*(98) = –2.09, *p* = 0.040], respectively. Pre-VSs expected itch was lower in the positive compared to the negative VSs group; [*t*(98) = 2.15, *p* = 0.034]. For the closed-label arm of studies 2 and 3, the positive VSs group scored lower on lie/social desirability compared to the negative VSs group; [*t*(101) = 3.24, *p* = 0.002].

### Simple Mediation: Effects of Verbal Suggestions on Mean Itch, as Mediated by Expectations

#### Open-Label Positive Verbal Suggestions Versus Neutral Instructions (Study 1)

**FIGURE 2:**
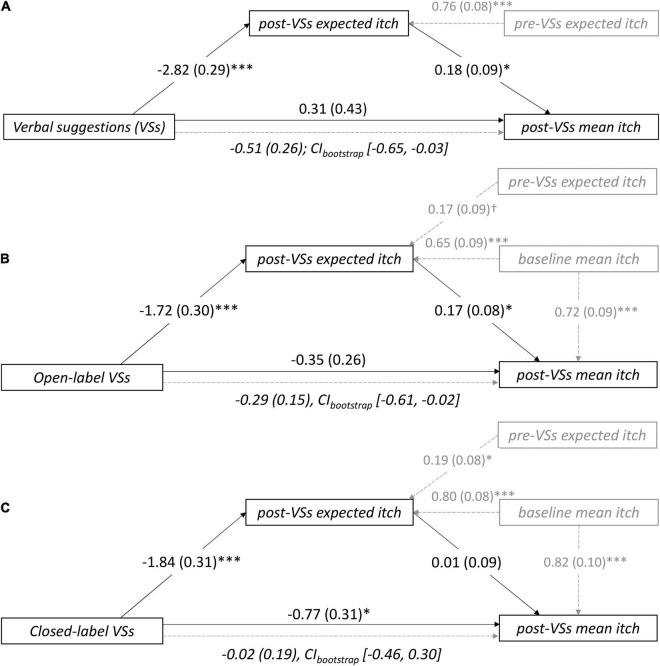
Unstandardized regression coefficients (SEM) for the mediation of the association between verbal suggestions (VSs) and post-VSs mean itch by itch expectations in **(A)** study 1, **(B)** the open-label arm of studies 2–3, and **(C)** the closed-label (i.e., concealed) arm of studies 2–3. The models were controlled for pre-VSs itch expectation **(A–C)** and baseline itch **(B,C)**. Note that c = (indirect) mediation effect; CI_bootstrap_ = bootstrapped confidence interval; ^†^*p* < 0.10; *p < 0.01; ***p < 0.001.

Mediation analysis (see Figure 1 for the conceptual model) revealed that positive VSs were significantly associated with lower expected itch compared to neutral instructions [path a_1_: *b*_X→M_ = –2.82, *SE* = 0.29, *p*<0.001; also described in Meeuwis et al. (2018)]. Within the model with positive VSs, lower post-VSs expected itch was significantly associated with lower post-VSs mean itch (path b_1_: *b*_M→Y_ = 0.18, *SE* = –0.09, *p* = 0.048). Positive VSs were not directly associated with lower mean itch (path c’: *b*_X→Y_ = 0.31, *SE* = 0.43, *p* = 0.47), however, a significant indirect association between positive VSs and lower mean itch was observed [path c: *b*_indirect_ = –0.51, *SE* = 0.26, 95% CI_bootstrap_ (–0.65, –0.03)]. This indicates that positive VSs indirectly reduced post-VSs mean itch, through mediation by expectation. Finally, lower pre-VSs expected itch was significantly associated with lower post-VSs expected itch [path e_1_: *b*_C→M_ = 0.76, *SE* = 0.08, *p* < 0.001; Figure 2A and Supplementary Table 3).

#### Open-Label Positive Versus Negative Verbal Suggestions (Studies 2 and 3)

Mediation analysis demonstrated that positive VSs reduced expected itch compared to negative VSs (path a_1_: *b*_X→M_ = –1.72, *SE* = 0.30, *p* < 0.001; findings of the separate studies are described in Meeuwis et al., 2019; Meeuwis et al., 2021). Lower post-VSs expected itch in turn was associated with lower post-VSs mean itch (path b_1_: *b*_M→Y_ = 0.17, *SE* = 0.08, *p* = 0.032). While no significant direct effect of VSs on mean itch was found (path c’: *b*_X→Y_ = –0.35, *SE* = 0.26, *p* = 0.19), again a significant indirect association between positive VSs and lower post-VS mean itch was observed [path c: *b*_indirect_ = –0.29, *SE* = 0.15, 95% CI_bootstrap_ (–0.61, –0.02)]. This shows that the effects of positive VSs versus negative VSs were mediated by post-VS expected itch under open-label conditions, with positive VSs being associated with lower mean itch than negative VSs. Finally, lower pre-VSs expected itch was marginally associated with lower post-VSs expected itch (path e_1_: *b*_C→M_ = 0.17, *SE* = 0.09, *p* = 0.054). Moreover, lower mean itch experienced during baseline significantly predicted lower post-VSs expected itch (path e_2_: *b*_C→M_ = 0.65, *SE* = 0.08, *p* < 0.001) and lower post-VSs mean itch (path e_3_: *b*_C→Y_ = 0.72, *SE* = 0.09, *p* < 0.001), respectively (Figure 2B and Supplementary Table 3).

#### Closed-Label Positive Versus Negative Verbal Suggestions (Studies 2 and 3)

Mediation analysis demonstrated that positive VSs reduced expected itch compared to negative VSs (path a_1_: b_X→M_ = –1.84, SE = 0.31, *p* < 0.001; findings of the separate studies are described in Meeuwis et al., 2019; Meeuwis et al., 2021). However, post-VSs expected itch in turn was not associated with post-VSs mean itch (path b_1_: b_M→Y_ = 0.01, SE = 0.09, *p* = 0.90). Instead, positive VSs were directly and significantly associated with lower post-VSs mean itch compared to negative VSs (path c’: b_X→Y_ = –0.77, *SE* = 0.31, *p* = 0.014). No significant indirect association between VSs and itch was found, which indicates that post-VSs expected itch did not mediate the effects of VSs on mean itch [path c: b_indirect_ = –0.02, *SE* = 0.19, CI_bootstrap_ (–0.46, 0.30)] in the closed-label context. Finally, lower pre-VSs expected itch was significantly associated with lower post-VSs expected itch (path e_1_: *b*_C→M_ = 0.19, *SE* = 0.08, *p* = 0.025). Moreover, lower mean itch experienced during baseline significantly predicted lower post-VSs expected itch (path e_2_: 0.80, *SE* = 0.09, *p* <0.001) and lower post-VSs mean itch (path e_3_: *b*_C→Y_ = 0.82, *SE* = 0.10, *p* < 0.001), respectively (Figure 2C and Supplementary Table 3).

### Conditional Process Analyses: Interindividual Differences in the Relation Between Verbal Suggestions, Expectations and Itch

#### Open-Label Positive Verbal Suggestions Versus Neutral Instructions (Study 1)

Conditional process analyses revealed no evidence for moderated mediation, which indicates that the expectation-mediated indirect effects of VSs on mean itch did not depend on interindividual differences in psychological traits or affective states (see Supplementary Table 4). A non-significant marginal first-stage interaction between VSs and extraversion was observed for expected itch (*p*_int_ = 0.086). *Post-hoc* probing of this interaction revealed that effects of VSs on expected itch were significant across all levels of extraversion though, and stronger when extraversion scores were higher (see Supplementary Figure 2). Other interindividual differences did not moderate the effects of VSs at either the first stage (post-VSs expected itch) or second stage (post-VSs mean itch) of the model, nor the effects of expected itch on mean itch at the second stage (all *p*_int_ ≥ 0.14; Supplementary Table 4). Across all moderated mediation models, lower pre-VSs expected itch predicted lower post-VSs expected itch (path e: range *b*_C→M_ = 0.74–0.76, all *SE* = 0.08, all *p* < 0.001). Direct associations between the psychological traits and affective states and outcomes are described in Supplementary Table 4.

#### Open-Label Positive Versus Negative Verbal Suggestions (Studies 2 and 3)

**FIGURE 3:**
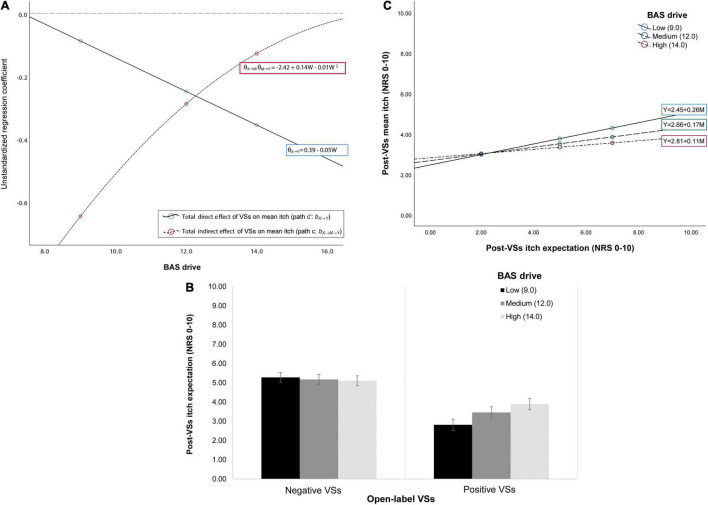
Depiction of the conditional indirect and direct effects of VSs on mean itch across low, medium and high levels of behavioral activation system (BAS) trait drive for the open-label arms of studies 2–3. **(A)** There was moderated mediation (depicted in **(A)** as the change in unstandardized regression coefficient magnitude for the effects of VSs on mean itch for low, medium and high levels of the moderator): the indirect (i.e., expectation-mediated) effects of VSs on mean itch (path c) changed depending on the level of BAS drive (i.e., the motivation to achieve goals). The effects of VSs on mean itch were significantly mediated by expectations in case of low drive to achieve goals (i.e., when BAS drive scores were low). When participants had high drive to achieve their goals (i.e., when BAS drive scores were high), expectations did not mediate the association between VSs and mean itch; instead, the direct effects of VSs on itch (path ‘c) tended to be stronger. This moderated mediation can also be explained as follows: **(B)** positive VSs were associated with lower itch expectation compared to negative VSs when BAS drive was lower (significant BAS drive × VSs interaction; depicted in **(B)** as mean itch expectation ± SEM for low, medium and high BAS drive levels); and **(C)** the association between lower itch expectation and lower post-VSs mean itch was stronger at low compared to high levels of BAS drive (depicted in **(C)** as simple regression slopes for each level of the moderator).

Conditional process analysis showed changes in the conditional indirect (i.e., expectation-mediated) effects of VSs on mean itch across different levels of the BAS drive trait. At low and medium levels of BAS-drive (i.e., when participants have lower drive to pursue their goals), the indirect effect of VSs on mean itch through expectations was larger than at high levels of BAS drive (i.e., when there is a high drive to pursue goals; Figure 3A). Moreover, the observed indirect effects were significant only at low and medium levels of BAS drive (i.e., bootstrapped 95%CI ≤ –0.04). *Post-hoc* pairwise contrasts confirmed moderated mediation, as the observed effects contrasted significantly for low, medium and high levels of this moderator (i.e., bootstrapped 95%CI ≥ 0.03; Supplementary Table 5). The model further inferred a non-significant marginal interaction between VSs and BAS drive for post-VSs expected itch (*p*_int_ = 0.075; see Figure 3B): effects of VSs on expected itch were stronger (i.e., expected itch was lower after positive VSs) when BAS drive was lower. The effects of expectations on mean itch were not moderated by BAS drive, although associations between expected itch and mean itch tended to be stronger for lower levels of BAS drive (Figure 3C). Direct effects of VSs on mean itch were not significantly moderated by BAS drive (both *p*_int_ > 0.18), but increases in effect magnitude could be observed when BAS drive scores were higher. Overall, the model shows that the effects of positive and negative VSs on mean itch may be more dependent on mediation by expectation when participants have generally lower drive to pursue their goals.

There was no evidence for moderated mediation in any of the models containing the other psychological traits or affective states (Supplementary Table 5). Some direct moderation effects were observed: the effects of VSs on expected itch were stronger at lower levels than at higher levels of BAS fun seeking (*p*_int_ = 0.015; Supplementary Figure 3). Body awareness moreover moderated the effects of VSs on expected itch (*p*_int_ = 0.047): the effects of VSs on expected itch were stronger for participants with lower body awareness (Supplementary Figure 4). Marginal non-significant trait x expected itch interaction effects on mean itch were observed for BAS reward responsiveness (*p*_int_ = 0.095) and the lie/social desirability scale (*p*_*int*_ = 0.083): the associations between post-VSs expected itch and mean itch were stronger when reward responsiveness was lower and when social desirability was higher (Supplementary Figures 5, 6). Finally, the association between pre-VSs expected itch and post-VSs expected itch ranged from marginal to significant (path e_1_: range *b*_C→M_ = 0.15–0.30, *SE* = 0.09–0.11, *p* = 0.007–0.097) across all moderated mediation models. Lower mean itch experienced during baseline iontophoresis was a significant predictor of post-VSs expected itch (path e_2_: range *b*_C→M_ = 0.37–0.66, *SE* = 0.09–0.14, all *p*< 0.01) and of post-VSs mean itch (path e_3_: range *b*_C→M_ = 0.66–0.73, *SE* = 0.09–0.11, all *p*< 0.001) across all models.

#### Closed-Label Positive Versus Negative Verbal Suggestions (Studies 2 and 3)

**FIGURE 4:**
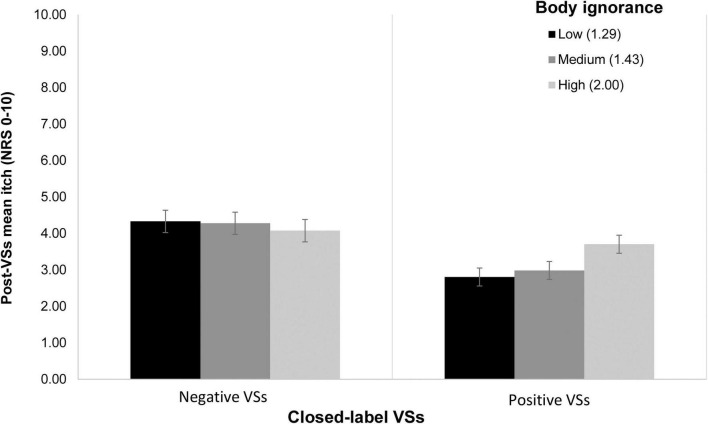
Depiction of the marginal moderation of the direct effects of VSs on mean itch by ignorance of bodily signals in the closed-label (i.e., concealed) arms of studies 2 and 3: at low and medium levels of body ignorance, effects of VSs on itch were larger, with participants in the positive VSs group reporting lower levels of itch and participants in the negative VSs group reporting higher levels of itch. When participants tended to ignore bodily signals to a high degree, effects of VSs on mean itch were non-significant. The associations are depicted in the figure as mean itch ± SEM for low, medium and high levels of body ignorance.

Conditional process analyses revealed no evidence for moderated mediation (Supplementary Table 6). A non-significant marginal moderation of the direct effects of VSs on mean itch by body ignorance was found (*p*_int_ = 0.072). Probing of this interaction revealed that at low and medium levels of body ignorance, positive VSs were significantly associated with lower mean itch compared to negative VSs (i.e., bootstrapped 95%CI ≤ –0.51). For high levels of body ignorance, effects of positive compared to negative VSs on mean itch were not significant (i.e., the bootstrapped 95%CI contained 0; Figure 4). Finally, the direct effect of VSs on post-VS expected itch was moderated by BAS fun seeking (i.e., the tendency to seek out pleasant stimuli) and BAS reward responsiveness (i.e., the sensitivity to rewarding stimuli), respectively (both *p*_int_ ≤ 0.031). *Post-hoc* probing of these moderation effects indicated that, in both models, positive compared to negative VSs resulted in lower expected itch when scores on the BAS subscale were low. When BAS scores were high, positive VSs were not associated with lower expected itch compared to negative VSs (Supplementary Figures 7, 8). Finally, the association between pre-VSs expected itch and post-VSs expected itch ranged from marginal to significant (path e_1_: range *b*_C→M_ = 0.15–0.38, *SE* = 0.08–0.14, *p* = 0.008–0.088) across all moderated mediation models. Lower mean itch experienced during baseline iontophoresis was a significant predictor of post-VSs expected itch (path e_2_: range *b*_C→M_ = 0.78–0.82, *SE* = 0.09–0.15, all *p*< 0.001) and of post-VSs mean itch (path e_3_: range *b*_C→M_ = 0.78–0.86, *SE* = 0.10–0.14, all *p*< 0.001) across all models.

## Discussion

The current work explored whether interindividual differences in psychological traits and affective states could modulate the formation of placebo and nocebo effects in histamine-induced itch by moderating either the direct effects of verbal suggestions on itch, or by moderating effects arising through mediation by conscious expectations. The results show that the effects of open-label verbal suggestions were predominantly mediated by (consciously rated) expectations, whereas for closed-label (i.e., concealed) suggestions, verbal suggestions directly modulated itch levels without involvement of conscious expectations. Sensitivity of the behavioral activation system (BAS), which is linked to reward responding, was associated with differences in the process of placebo and nocebo responding. This is evidenced by the various significant moderated mediation and moderation effects found for BAS-associated trait scales across the three studies. In particular, the effects of open-label verbal suggestions on itch were more strongly mediated by conscious expectations when BAS trait drive (i.e., the motivation to pursue one’s goals) was lower. In addition, the extent to which individuals pay attention to and ignore bodily signals was related to placebo and nocebo effects: participants who have a higher tendency to ignore bodily symptoms tended to respond more strongly to the positive or negative verbal suggestions. There was no evidence that other interindividual differences, for instance in optimism, neuroticism, or worrying, modulated placebo and nocebo responding to itch.

The current work illustrates how conscious expectations may contribute to placebo and nocebo responding to open-label as well as closed-label verbal suggestions for itch across a relatively large sample of healthy volunteers. To our knowledge, it is the first work that explores how interindividual differences may shape the response to these suggestions and simultaneously takes into account that this influence may be via indirect (i.e., expectation-mediated) pathways. Notably, the role of conscious expectations appeared limited for the closed-label, or concealed, arm of studies 2 and 3. Instead, verbal suggestions directly influenced the experience of itch. Some studies show that conscious expectations may not be needed for placebo or nocebo effects to occur (Jensen et al., 2012; Jensen K. B. et al., 2015; Bajcar et al., 2020; Colloca et al., 2020). The current findings for closed-label suggestions are in line with this previous evidence. On the other hand, the findings for the open-label suggestions show that the effects of these suggestions were predominantly mediated by conscious expectations. Because the research area of open-label placebo is relatively new, much less is known about the mechanisms of these specific placebo effects, or the role that expectations may have in shaping them. A prior experimental study with healthy volunteers shows that expectations about how well placebo pills would work for the participant can influence open-label placebo effects, independent of the actual dose or adherence to placebo treatment (El Brihi et al., 2019). Other studies moreover show that the rationale provided by the researchers for open-label placebo influences the magnitude of open-label placebo effects for patients, which could indicate that conscious expectations play a role in these effects (Locher et al., 2017; Schaefer et al., 2018; Leibowitz et al., 2019). The current findings are in line with these studies. Notably, open-label placebo has been found to improve outcomes for patients, even when they were skeptical or did not expect to experience benefits (Kaptchuk, 2018; Kaptchuk and Miller, 2018), which suggests that factors other than expectations may also elicit these effects. More research is needed to examine under which circumstances and to which extent expectations can contribute to open-label placebo effects.

With regard to the interindividual differences that predict placebo and nocebo effects, traits related to the BAS were found to moderate the effects of positive and negative suggestions on expectations and itch in the open-label arm of studies 2 and 3. BAS is a motivational system that reflects an individual’s sensitivity to stimuli of reward and punishment (Carver and White, 1994; Dierickx et al., 2021). Higher BAS-associated traits and higher sensitivity to rewards have been associated with increased pain experience (Day et al., 2019; Sánchez-Rodríguez et al., 2021; Turner et al., 2021), and notably, with enhanced placebo analgesia as well (Schweinhardt et al., 2009; Yu et al., 2014; De Pascalis and Scacchia, 2017). In the current work, the BAS traits drive, reward responsiveness, and fun seeking all influenced expectations, and consequently, open-label placebo and nocebo effects in itch to some extent. Notably, the indirect effects of positive and negative suggestions on mean itch were larger when the BAS trait “drive” was lower. While this seems contrary to the existing literature at first, these findings could in fact reflect that the process by which placebo and nocebo effects are formed differs depending on an individual’s sensitivity to rewards. The significant moderated mediation that we found for the BAS trait “drive” supports this in particular. When participants indicated low drive to pursue their goals, the effects of open-label verbal suggestions on itch were more strongly mediated by expectations. In contrast, the direct effects of verbal suggestions increased in magnitude when BAS drive was higher. Although this increase in magnitude was non-significant in the current work, this would be in line with findings of prior studies (Schweinhardt et al., 2009; De Pascalis and Scacchia, 2017). Similar patterns could be noticed in the findings for BAS “reward processing” (i.e., trait reflecting sensitivity to rewards) and BAS “fun seeking” (i.e., reflecting the tendency to seek out novel or rewarding stimuli). Though moderated mediation was absent, these two scales did moderate some of the single pathways in the model (for instance, the effects of suggestions on expectations). Taken together, these findings show that for individuals who have low BAS (i.e., low sensitivity to rewards), changes in conscious expectations may be necessary to elicit placebo and nocebo effects. For individuals who have a highly sensitive BAS, suggestions could influence itch regardless of what they expect to happen. This implies that it could be relevant to adjust communication strategies in clinical practice depending on a patient’s BAS: for those with low BAS, it may be more prudent to maximize positive expectations about treatment for itch.

High BAS has often been associated with higher extraversion (e.g., in Heubeck et al., 1998; Smits and Boeck, 2006). Notably, while BAS was associated with placebo and nocebo responding in the current work, extraversion was not. This is not in line with previous work that links extraversion with placebo responding (Beedie et al., 2008; Kelley et al., 2009), though generally findings for extraversion and placebo responding are mixed (Kern et al., 2020). Extraversion did modulate the effects of suggestions on expectations, though. Potentially, this modulation may not have been large enough to result in detectable differences in placebo or nocebo responding in actual itch experience. Moreover, according to Gray’s original theory on reinforcement sensitivity, extraversion stems from a combination of high BAS and low BIS (Gray, 1970; in Knyazev et al., 2004), and BIS has not been associated with placebo or nocebo responding so far, including in the current work. This may speculatively explain why BAS modulated placebo and nocebo effects in the current work, but extraversion did not. Alternatively, variance in extraversion could have been too small to detect associations with placebo or nocebo responding, as young and healthy student volunteers were predominantly included here. Finally, psychological traits or affective states may interact among themselves whilst influencing health outcomes. To illustrate, interactions between the Big Five personality traits have been found to predict wellbeing and mood (McFatter, 1994; Merz and Roesch, 2011). Interindividual differences in a single trait as such may not influence outcomes insomuch, but a specific combination of traits might. While these between-trait interactions are outside of the scope of the current work, future studies could consider, for instance, to use multiplicative moderation analyses to detect whether interactions among moderators may influence placebo or nocebo responding.

It should be noted that, while previous studies investigated placebo effects and BAS exclusively (without looking into nocebo effects), we compared open-label positive suggestions with either neutral instructions or negative suggestions. The results show that BAS traits did not moderate the effects of positive suggestions versus neutral instructions—but rather, that they significantly modulated the effects of positive versus negative suggestions. Thus, alternatively, our findings could also indicate that the involvement of reward processing is different in placebo compared with nocebo effects. This would be in line with recent evidence that shows that activity in the ventral striatum differs between placebo and nocebo effects, likely because placebo responding may be a form of reward processing, whereas nocebo responding may engage aversive networks in the brain (Fu et al., 2021). BAS traits have been found to consistently correlate with activity of this brain region in response to positive stimuli (see for example, Kennis et al., 2013). Moreover, there is evidence that placebo analgesia activates the reward system in the brain, whereas nocebo hyperalgesia may inhibit this network (Shi et al., 2021). Our findings that when BAS-drive trait is low, placebo versus nocebo responding is more dependent on expectation change than when this trait is highly present could reflect these differential responses of the reward system, although this needs to be confirmed by fMRI research. Brain imaging studies for placebo and nocebo effects have so far been conducted predominantly in pain. To this date, only two imaging studies have been published that explore the brain areas involved in nocebo effects for itch (Napadow et al., 2015; van de Sand et al., 2018), and none have studied the brain mechanisms of placebo effects in itch yet. Brain areas that have been found to be involved in nocebo effects in itch are also involved in motivational processing (Napadow et al., 2015). Moreover, interaction between cortex and periaqueductal gray (PAG) was enhanced in nocebo responding in itch (van de Sand et al., 2018). Activation of the PAG in particular has been implicated in descending pain control and reward function (Becerra et al., 2001), but is central to itch processing as well (Najafi et al., 2021). Interestingly, PAG deactivation deriving from reward system activation following scratching has been found to relieve itch, which may suggest distinct mechanisms for itch compared to pain relief (Papoiu et al., 2013). Speculatively, this could mean that the neurophysiological mechanisms of placebo effects differ between itch and pain as well.

Marginal moderation of the direct effects of closed-label positive and negative suggestions on itch by body ignorance was found. Participants who indicated that they tended to ignore signals of their body showed larger placebo and nocebo responses to the verbal suggestions that were provided. Potentially, these individuals’ responses and experiences may be guided to a larger extent by external signals rather than internal ones. Alternatively, individuals who indicated that they tend to be aware of what they experience in their body may be guided more by internal signals and less so by external information. There is some evidence that more self-aware people experience less arousal following a placebo intervention (Gibbons et al., 1979). Training patients to accurately evaluate and report pain levels based on internal rather than external cues has also been found to reduce placebo responses in chronic low back pain (Erpelding et al., 2020). The current findings are in line with this. However, it should be noted that awareness and ignorance of bodily signals in the current study were assessed through self-report, and may as such reflect a conviction that people have (i.e., they believe that they ignore their symptoms) and not a particular skill set. It may be relevant to further investigate whether self-reported versus actual skill in recognizing bodily signals influences placebo and nocebo responding in itch. Moreover, the current study compared placebo and nocebo effects elicited by suggestions. Future research may aim to investigate whether ignorance of bodily symptoms contributes equally to placebo and nocebo effects, for instance by comparing these effects with a neutral control condition. Training individuals to evaluate itch accurately may be particularly relevant for nocebo effects—in theory, such a training could be used to reduce the occurrence of these effects in clinical practice.

The current work shows that other, more general psychological traits, such as optimism, neuroticism, or worrying were not associated with placebo and nocebo responding to verbal suggestions in itch. Although some direct moderation effects were found in the current work, for instance of traits and suggestions on expectations, these were not actually associated with itch experience. This is in line with studies that show that these traits do not predict placebo or nocebo responses (Corsi and Colloca, 2017; Gillving et al., 2020; Kern et al., 2020), but contradicts several studies that do report such associations (e.g., that optimism can predict placebo responding: see Geers et al., 2005, 2007, 2010; Morton et al., 2009; Darragh et al., 2014; Corsi et al., 2016; Zhou et al., 2019). These discrepancies between study findings may be attributable to differences in methodology, or to differences in the type of symptoms that were assessed (i.e., pain versus itch). In addition, the contribution of these interindividual differences to placebo and nocebo effects may change depending on the manner in which placebo and nocebo effects were induced. Identifying which interindividual differences can contribute to placebo and nocebo responding, and which cannot, remains important in order to develop strategies aimed at maximizing placebo effects and minimizing nocebo effects in clinical practice (Evers et al., 2021). Future research could, for example, assess whether the factors that are relevant for shaping placebo and nocebo effects differ depending on the type of mechanisms that elicit these effects. If we know which interindividual differences are relevant for which mechanisms, we will be able to better predict for whom interventions or a treatment rationale aimed at optimizing expectations would be helpful, for instance, and as such be able to optimize treatment in clinical practice.

Innovative statistical methods were used to obtain detailed and mechanistic information about the potential influence of interindividual differences on both open-label and closed-label placebo and nocebo effects in itch. Other strengths of the current work include the increase in power for the analyses that was obtained by combining data of the three studies, and the similarity in the assessed psychological traits and affective states across studies. Some limitations need to be addressed. First, the methodology varied across the analyzed studies, and it cannot be ruled out that some variations in the current findings could be attributed to these between-study differences. For instance, in studies 2 and 3 positive and negative verbal suggestions were compared to each other, but not to a control group. Findings in those studies likely describe differences between placebo and nocebo responders, whereas those in study 1 describe placebo responders only. Second, the main aim of this paper was to explore associations between interindividual differences, expectations, and itch experience following verbal suggestions, and as such the findings need to be seen as hypothesis-generating. A large number of statistical tests were performed to achieve this, which may have increased the number of chance findings. Nonetheless, some measures were taken to prevent over-reporting of chance findings. For instance, a bootstrap-based method was used to analyze mediation and conditional processes. Bootstrapping can improve the accuracy of confidence intervals (Preacher and Hayes, 2008; Hayes, 2009, 2015). Third, the reported effects tended to be small. Moreover, some between-group differences in psychological traits and affective states were observed, for instance between the open-label positive and negative VSs groups of studies 2 and 3: the positive VSs group scored higher on BAS drive trait. While bootstrapping generally can handle asymmetric sampling well (Preacher and Hayes, 2008; Hayes, 2009, 2015), some caution may be needed in interpreting these findings and, ideally, they would need to be replicated by future studies. Finally, a relatively homogenous study sample of young, predominantly female, and healthy student volunteers was used. This may have influenced findings, for instance, by impacting the diversity in the assessed interindividual differences. Generalization of the findings to the general population should be done carefully and in light of the assessed study sample.

In short, the current study explored whether interindividual differences modulated how placebo and nocebo effects are shaped in histamine-induced itch. Moderation of both the direct and indirect (expectation-mediated) effects of positive and negative verbal suggestions were tested. The results indicate that the effects of open-label positive and negative suggestions on itch may be more dependent on mediation by expectations, whereas closed-label (i.e., concealed) suggestions influenced itch directly. Moreover, the findings show that the process by which the positive and negative suggestions influenced itch can change depending on BAS sensitivity: for individuals who have low BAS (i.e., low sensitivity to rewards), the effects of suggestions were mediated more strongly by expectations. In addition, high ignorance of bodily signals was marginally associated with increased placebo and nocebo responding to verbal suggestions. Finally, there was no evidence that other interindividual differences, for instance in optimism, neuroticism or worrying, modulated placebo and nocebo responding in itch. Overall, the findings contribute to the growing collection of studies that identify factors associated with placebo and nocebo effects. Innovative statistical methods were used to obtain detailed mechanistic information about the potential influence of interindividual differences on how placebo and nocebo effects were formed. If we can increase our understanding of these processes, we may then use this knowledge to develop strategies aimed at maximizing placebo effects in clinical practice.

## Data Availability Statement

The data analyzed in this study is subject to the following licenses/restrictions: The datasets analyzed for this study are available on request to the corresponding author. Requests to access these datasets should be directed to corresponding author.

## Ethics Statement

The studies involving human participants were reviewed and approved by the Medical Ethics Committee, Leiden University Medical Center, Leiden, Netherlands. The patients/participants provided their written informed consent to participate in this study.

## Author Contributions

SM undertook the statistical analyses and wrote the first draft of the manuscript. All authors commented on the manuscript and approved of the final version of the manuscript.

## Conflict of Interest

The authors declare that the research was conducted in the absence of any commercial or financial relationships that could be construed as a potential conflict of interest.

## Publisher’s Note

All claims expressed in this article are solely those of the authors and do not necessarily represent those of their affiliated organizations, or those of the publisher, the editors and the reviewers. Any product that may be evaluated in this article, or claim that may be made by its manufacturer, is not guaranteed or endorsed by the publisher.

## Abbreviations

BAS: Behavioral Activation System
BIS: Behavioral Inhibition System
NRS: Numeric Rating Scale
VSs: verbal suggestions.
