# Supplementary material for: Associations Between Interindividual Differences, Expectations and Placebo and Nocebo Effects in Itch
Source: Front Psychol. 2021 Dec 13;12:781521. doi: 10.3389/fpsyg.2021.781521 (PMC8711701; doi:10.3389/fpsyg.2021.781521)
Supplement: Supplementary file 1 [file Data_Sheet_1.pdf]

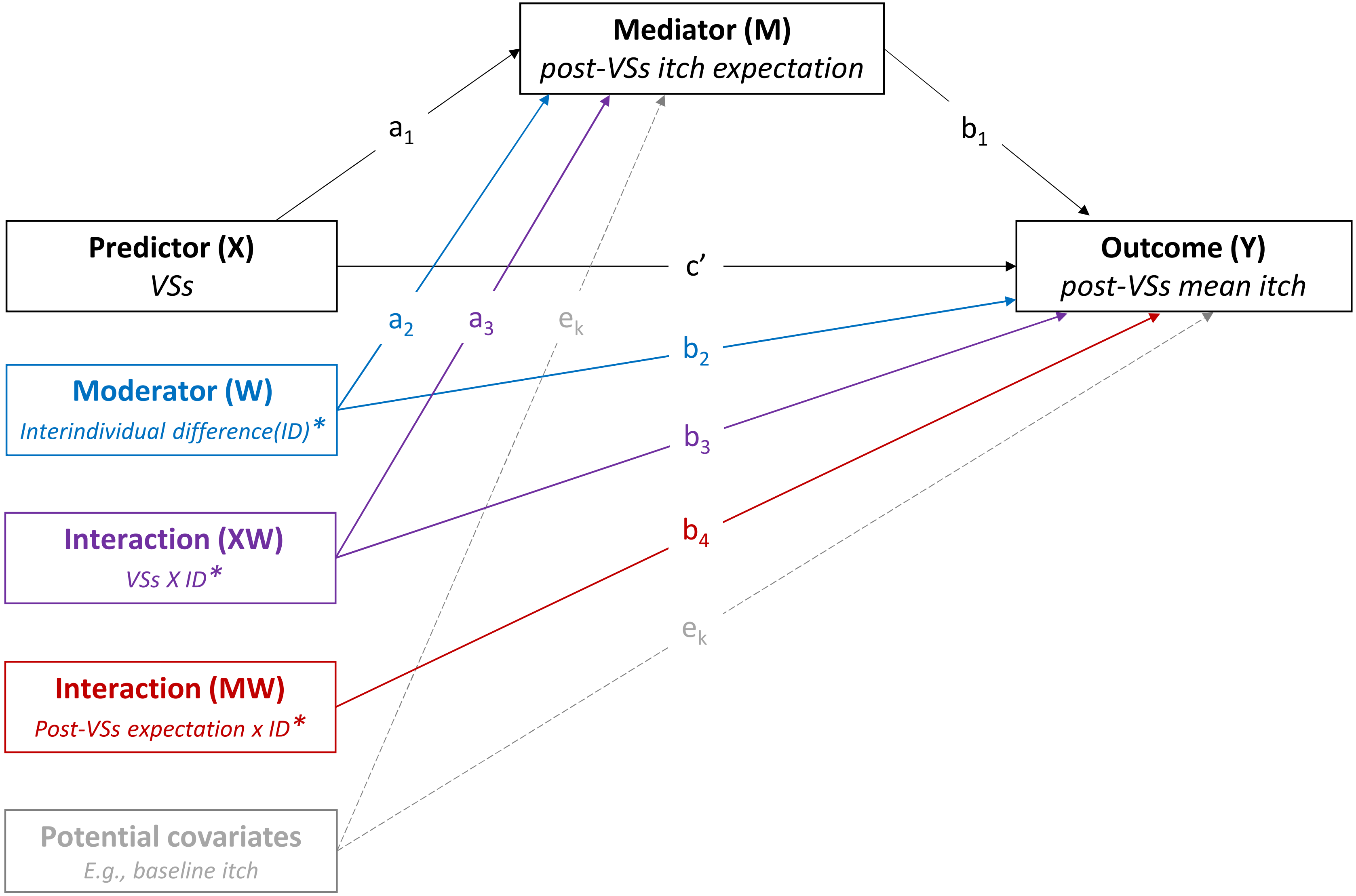


**Supplementary Figure 1**. Statistical representation of the first- and second stage dual moderated mediation model (model 59; Hayes, 2017).

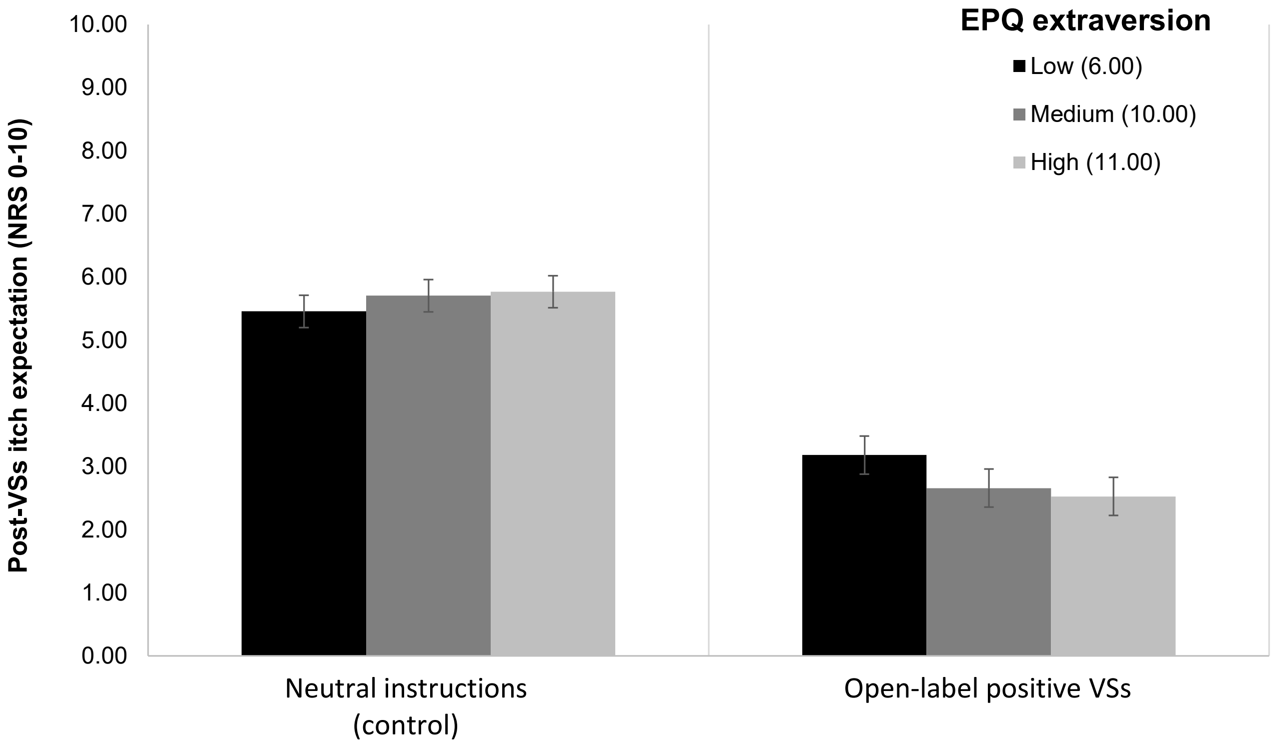


**Supplementary Figure 2.** The effects of open-label positive verbal suggestions (VSs) versus neutral control instructions on post-VSs expected itch were marginally moderated by extraversion in study 1. When extraversion scores were high, participants in the VSs group expected low itch in comparison with participants in the neutral instruction group, who expected high itch. At lower levels of extraversion, the effects of VSs compared to neutral instructions were smaller. Effects are plotted in the figure as mean (SEM) for low, medium and high levels of the moderator.

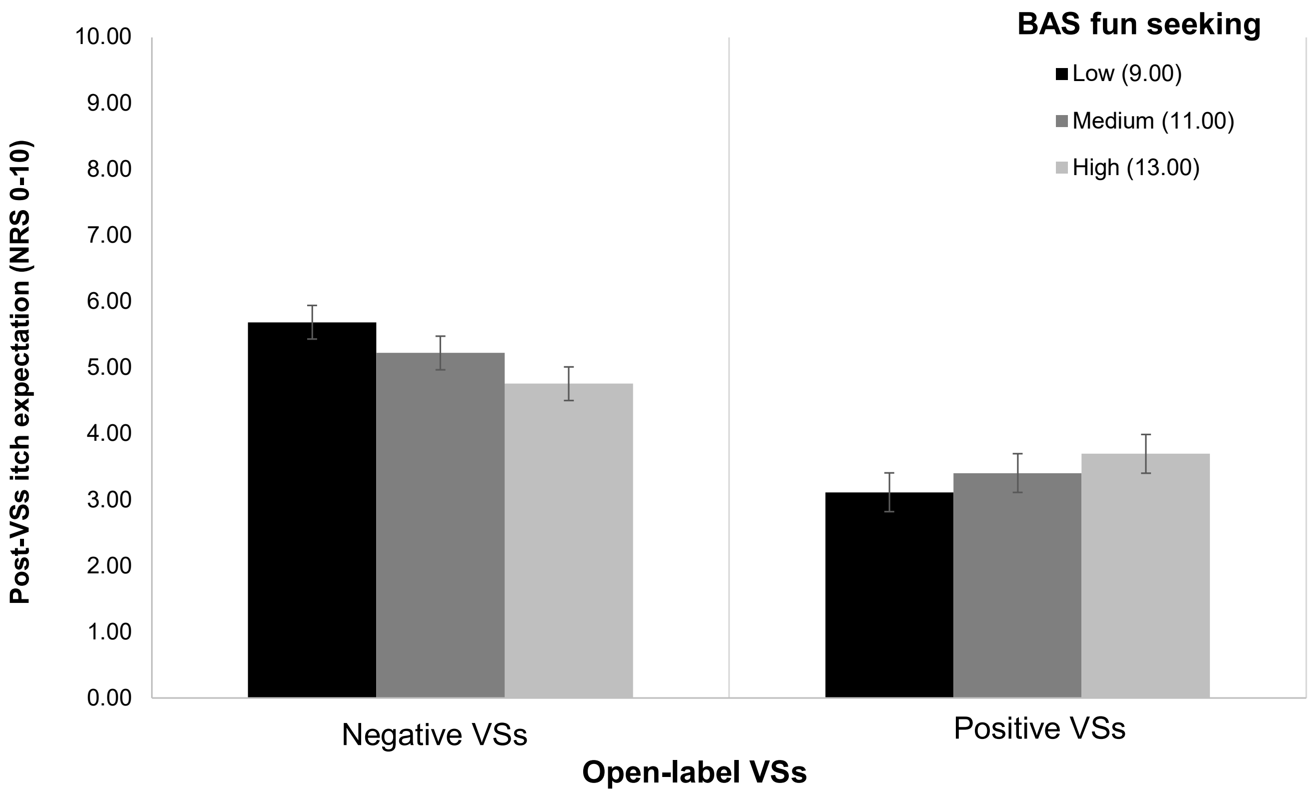


**Supplementary Figure 3.** The effects of open-label positive and negative VSs on post-verbal suggestions (VSs) expected itch were significantly moderated by the behavioural activation system (BAS) subscale 'fun seeking' (i.e., the tendency to seek out pleasant stimuli; study 2+3). When scores on this trait were low, participants in the negative VSs group expected high itch in comparison with participants in the positive VSs, who expected low itch. At higher levels of BAS fun seeking, effects of VSs across groups were smaller. Effects are plotted as mean (SEM) for low, medium and high levels of the moderator.

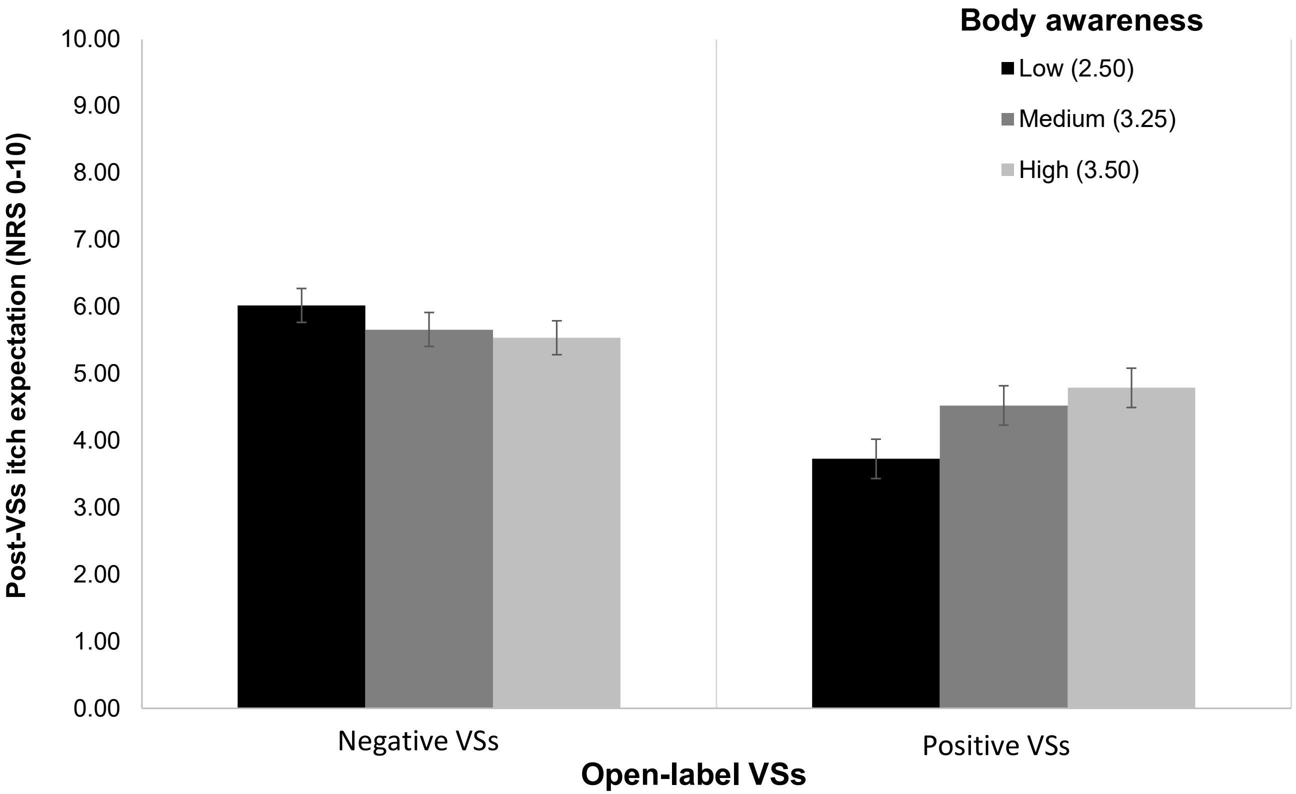


**Supplementary Figure 4.** The effects of open-label positive and negative VSs on post-verbal suggestions (VSs) itch expectation were significantly moderated by self-assessed body awareness (study 2+3): when participants indicated that their awareness of bodily signals scores was low, they expected high itch in the negative VSs group, in comparison with the positive VSs, where they expected low itch. At higher levels of body awareness, effects of VSs across groups were smaller. Effects are plotted as mean (SEM) for low, medium and high levels of the moderator.

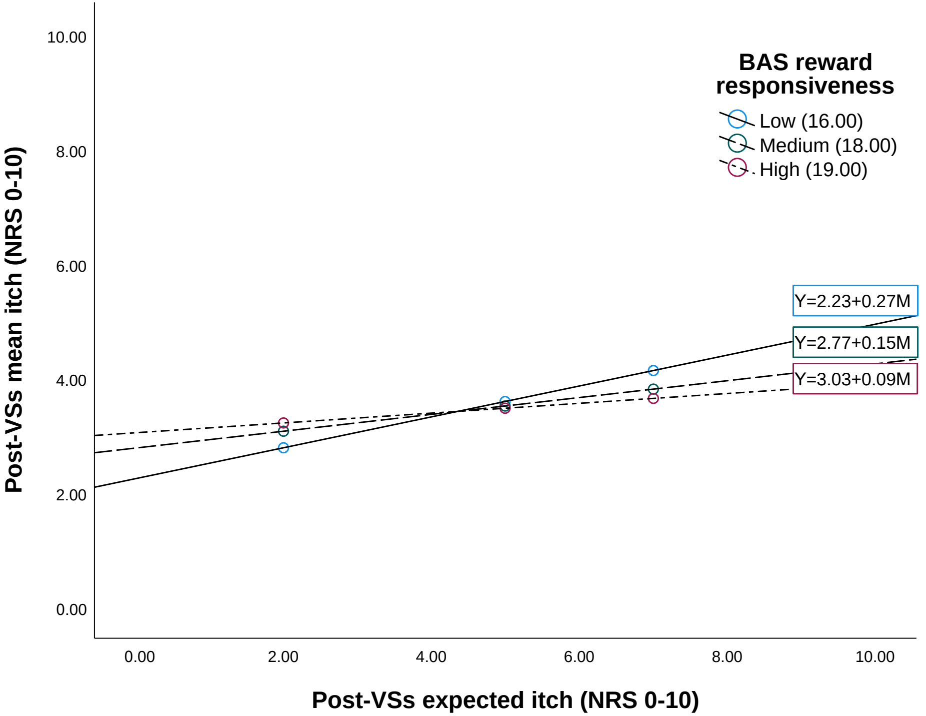


**Supplementary Figure 5.** Associations between post-VSs expected itch and post-VSs mean itch during histamine iontophoresis differed marginally across levels of BAS reward responsiveness (i.e., the tendency to react strongly to rewarding stimuli) for the open-label context (study 2+3). When BAS reward responsiveness was low, associations between expected itch and mean itch were stronger than when BAS reward responsiveness was high (as evidenced by a significant BAS * expected itch interaction effect).

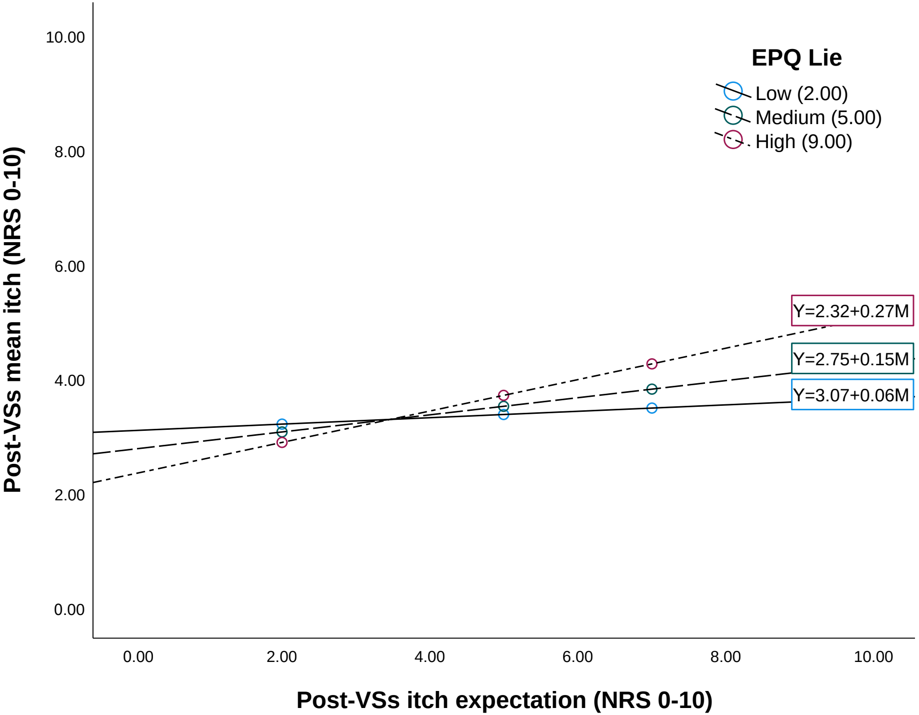


**Supplementary Figure 6.** Associations between post-VSs expected itch and post-VSs mean itch during histamine iontophoresis differed marginally across levels of social desirability (i.e., EPQ lie; the tendancy to answer in a socially desirable manner) for the open-label context (study 2+3). When social desirability was high, associations between expected itch and mean itch were stronger than when social desirability was low (as evidenced by a significant EPQ lie * expected itch interaction effect).

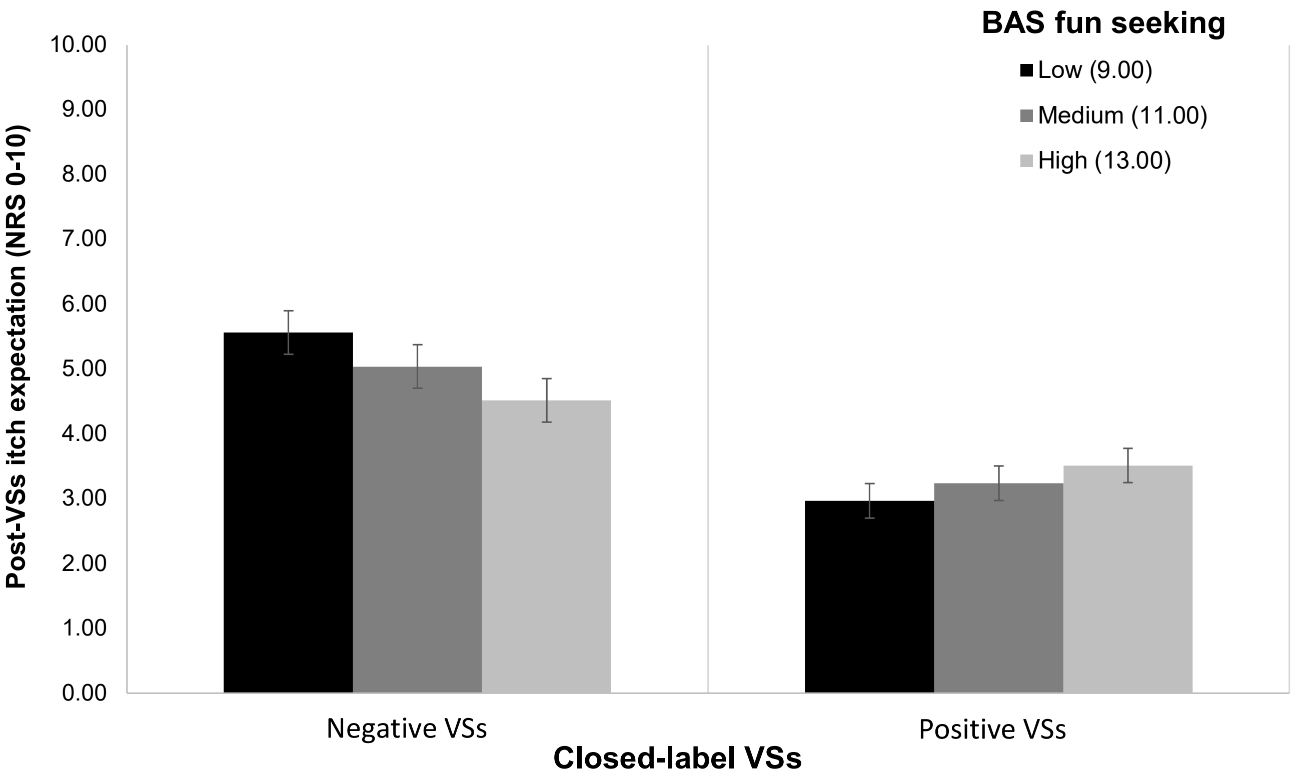


**Supplementary Figure 7**. The effects of closed-label (i.e., concealed) positive and negative VSs on post-verbal suggestions (VSs) itch expectation were significantly moderated by the behavioural activation system (BAS) subscale 'fun seeking' (i.e., the tendency to seek out pleasant stimuli; study 2+3): when scores on this trait were low, participants in the negative VSs group expected high itch in comparison with participants in the positive VSs, who expected low itch. At higher levels of BAS fun seeking, effects of VSs across groups were smaller. Effects are plotted as mean (SEM) for low, medium and high levels of the moderator.

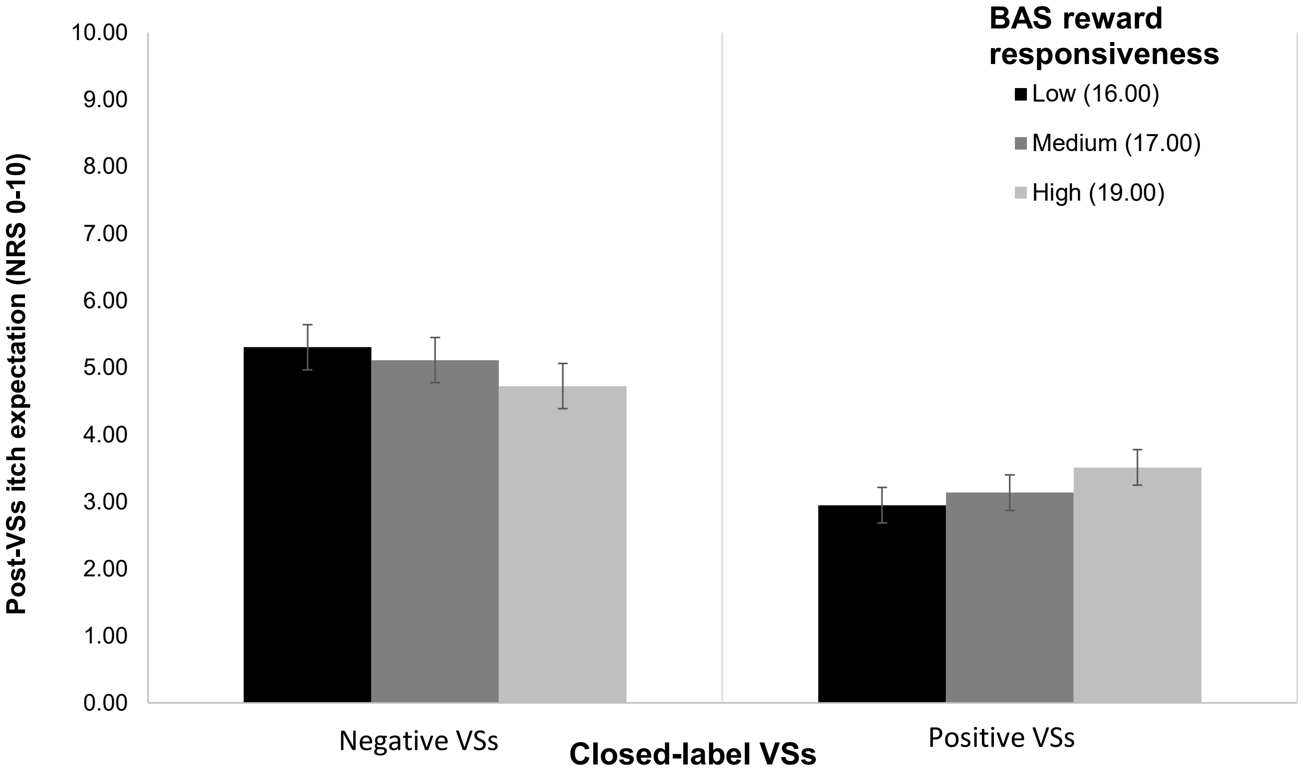


**Supplementary Figure 8**. The effects of closed-label (i.e., concealed) positive and negative VSs on post-verbal suggestions (VSs) itch expectation were significantly moderated by the behavioural activation system (BAS) subscale 'reward responsiveness' (i.e., the tendency to react strongly to rewarding stimuli; study 2+3): when scores on this trait were low, participants in the negative VSs group expected high itch in comparison with participants in the positive VSs, who expected low itch. At higher levels of BAS reward responsiveness, effects of VSs across groups were smaller. Effects are plotted as mean (SEM) for low, medium and high levels of the moderator.
