# Supplementary material for: Associations Between Interindividual Differences, Expectations and Placebo and Nocebo Effects in Itch
Source: Front Psychol. 2021 Dec 13;12:781521. doi: 10.3389/fpsyg.2021.781521 (PMC8711701; doi:10.3389/fpsyg.2021.781521)
Supplement: Supplementary file 2 [file Data_Sheet_2.pdf]

Supplementary Table 1. Similarities and differences between the studies included in the paper.

		**Study 1.**	**Study 2.**	**Study 3.**
Participants	Sample description	Healthy volunteers	Healthy volunteers	Healthy volunteers
	Sample size	n = 92	n = 92	n = 112
Study design	Experimental groups	1) Open-label positive VSs<br>2) Neutral control	1) Open-label positive VSs<br>2) Closed-label positive VSs<br>3) Open-label negative VSs<br>4) Closed-label negative VSs	1) Open-label positive VSs<br>2) Closed-label positive VSs<br>3) Open-label negative VSs<br>4) Closed-label negative VSs
Verbal suggestions*	VSs core content	"*This test will elicit little itch*"	"*Application of this tonic will decrease/increase itch during this test*"	"*Application of this transdermal patch will, as a side effect, decrease/increase itch during this test*"
	Open-label instruction core content	*"Telling you this test will elicit little itch will result in little itch being experienced"*	"*This tonic is actually a sham treatment. Merely suggesting to you that it is effective will most likely result in experiencing a decrease/increase in itch*"	"*This tonic is actually a sham treatment. Merely suggesting to you that it is effective will most likely result in experiencing a decrease/increase in itch. This is due to the brain responding to information about a treatment in the same manner as to actual treatment*"
Itch induction	Method	Histamine iontophoresis	Histamine iontophoresis	Histamine iontophoresis
	Duration	2.5 minutes	2.5 minutes	2.5 minutes
	Itch NRS	0 ('*no itch*') to 10 ('*worst itch ever experienced*')	0 ('*no itch*') to 10 ('*worst itch imaginable*')	0 ('*no itch*') to 10 ('*worst itch imaginable*')
	Timing of measurement during iontophoresis	Every 30 seconds	Continuously	Every 30 seconds
	Measurement of mean itch (*main outcome*)	YES (immediately after iontophoresis)	YES (immediately after iontophoresis)	YES (immediately after iontophoresis)
	Baseline iontophoresis included in study	NO	YES (assessed in separate session, 1 wk earlier)	YES (assessed within same session)
Expectations	Itch expectation NRS	0 ('no itch') to 10 ('worst itch ever experienced')	0 ('no itch') to 10 ('worst itch imaginable')	0 ('no itch') to 10 ('worst itch imaginable')
	Timing of post-VS expectation measurement	Immediately after VSs	Immediately after VSs	Immediately after VSs
	Baseline measurement included in study	YES (pre-VSs)	YES (pre-baseline iontophoresis)	YES (pre-baseline iontophoresis)
Questionnaires	Demographics	Sex	Sex	Sex
	Interindividual differences that were assessed in the study	Neuroticism & extraversion[A]; optimism[B]; distress[C]; perceived stress[D]; worrying[E]; BIS/BAS subscales 'drive', 'fun seeking', 'reward responsiveness', & 'behavioural inhibition'[F]	Neuroticism, extraversion, lie/social desirability[A]; optimism[B]; BIS/BAS subscales 'drive', 'fun seeking', 'reward responsiveness', & 'behavioural inhibition'[F]	Neuroticism, extraversion, lie/social desirability[A]; optimism[B]; BIS/BAS subscales 'drive', 'fun seeking', 'reward responsiveness', & 'behavioural inhibition'[F]; body attention, ignorance, & awareness[G]
	Timing of measurement	Online, pre-session	In the lab, pre-baseline iontophoresis	In the lab, pre-baseline iontophoresis

Note. * The content of the verbal suggestions and the open-label explanations of placebo/nocebo effects has been summarized and paraphrased here. For the full content of these instructions, please see our previous publications: Meeuwis et al., 2018, Meeuwis et al., 2019, Meeuwis et al., 2021.

BIS = behavioral inhibition system ; BAS = behavioural activation system; NRS = Numeric Rating Scale; VSs = verbal suggestion

Interindividual differences in psychological traits and affective states were assessed by [A] the Eysenck Personality Questionnaire – Revised Short Scales (EPQ-RSS; Eysenck & Eysenck, 1975), [B] the Life Orientation Test – Revised (LOT-R; Scheier et al., 1994), [C] the Hospital Anxiety and Depression Scales (HADS, Zigmond & Snaith, 1983), [D] the Perceived Stress Scale (PSS, Cohen et al., 1983),[E] the Penn State Worry Questionnaire (PSWQ, Meyer et al., 1990), [F] the Behavioral Inhibition System (BIS) / Behavioral Activation System (BAS) scales (BIS/BAS, Carver & White, 1994), and [G] the Body Attention, Ignorance, and Awareness Scale (BAIAS; van Beugen et al., 2015).

Supplementary Table 2. Overview of demographic factors, individual traits, and expectation and itch scores, including p-values of differences on these variables between groups.

	Study 1			Study 2 and 3					
				Open-label			Closed-label		
	Open-label positive VS (n = 46)	Neutral control (n = 46)	*p*	Positive VS (n = 49)	Negative VS (n = 51)	*p*	Positive VS (n = 51)	Negative VS (n = 52)	*p*
Age	21.39 ± 2.04	21.13 ± 1.85	.52	21.90 ± 2.84	22.02 ± 3.02	.84	22.06 ± 2.54	21.44 ± 2.42	.21
Sex [male]	10 (21.7%)	7 (15.2%)	.59	8 (16.3%)	8 (15.7%)	.57	9 (17.6%)	9 (17.3%)	.58
Interindividual differences									
Neuroticism	3.02 ± 2.88	2.91 ± 2.27	.84	4.53 ± 2.79	3.47 ± 2.38	.043	4.39 ± 2.91	4.37 ± 2.77	.96
Extraversion	8.91 ± 2.76	8.59 ± 2.36	.55	8.90 ± 2.85	9.18 ± 2.83	.63	9.02 ± 2.98	9.10 ± 2.82	.89
Lie/social desirability	-	-	-	4.90 ± 2.89	5.92 ± 3.12	.092	4.39 ± 2.67	6.02 ± 2.43	.002
Optimism	15.70 ± 3.02	16.72 ± 3.10	.11	15.47 ± 3.80	16.49 ± 3.66	.17	15.96 ± 4.09	16.25 ± 4.01	.72
Distress	7.13 ± 4.47	6.24 ± 3.94	.31	-	-	-	-	-	-
Perceived stress	12.39 ± 5.99	12.09 ± 4.85	.79	-	-	-	-	-	-
Worrying	41.35 ± 11.77	43.54 ± 11.76	.37	-	-	-	-	-	-
BAS drive	11.35 ± 2.30	11.43 ± 2.34	.86	12.08 ± 1.81	11.12 ± 2.70	.040	11.00 ± 2.32	11.08 ± 2.19	.86
BAS fun seeking	10.96 ± 2.03	11.11 ± 1.97	.72	11.43 ± 1.94	11.04 ± 1.93	.32	10.84 ± 2.08	10.87 ± 1.91	.96
BAS reward responsiveness	17.50 ± 1.84	17.63 ± 2.00	.75	17.57 ± 1.77	17.31 ± 1.59	.45	17.47 ± 1.75	17.25 ± 1.77	.53
Behavioral inhibition	19.57 ± 4.34	20.02 ± 3.72	.59	21.29 ± 3.39	20.43 ± 3.76	.24	20.92 ± 3.86	20.73 ± 3.67	.80
Body ignorance [a]	-	-	-	1.72 ± 0.36	1.55 ± 0.38	.089	1.59 ± 0.38	1.60 ± 0.43	.96
Body awareness [a]	-	-	-	3.06 ± 0.51	3.02 ± 0.48	.73	3.14 ± 0.35	2.92 ± 0.57	.081
Body attention [a]	-	-	-	2.44 ± 0.49	2.43 ± 0.48	.95	2.59 ± 0.51	2.51 ± 0.59	.63
Expectation ratings [b]									
Pre-VS expectation	4.84 ± 1.65	5.19 ± 1.83	.34	4.83 ± 1.89	5.61 ± 1.77	.034	5.21 ± 1.95	4.63 ± 1.91	.13
Post-VS expectation	2.69 ± 2.02	5.73 ± 1.72	< .001	3.35 ± 2.06	5.33 ± 1.81	< .001	3.41 ± 1.89	4.89 ± 2.43	.001
Histamine iontophoresis [b]									
Baseline mean itch	-	-	-	3.42 ± 1.62	3.60 ± 1.83	.59	3.74 ± 1.79	3.42 ± 1.81	.37
Post-VS mean itch	3.23 ± 1.72	3.46 ± 1.51	.50	3.00 ± 1.71	3.81 ± 1.96	.030	3.17 ± 1.77	3.69 ± 2.20	.19

Note. P-values were derived from independent sample t-tests for continuous variables. For sex, p-values were calculated using Chi-square tests. [a] n = 28 for all groups with the exception of the closed-label positive VS group (n=27). [b] As published in Meeuwis et al., 2018, Meeuwis et al., 2019, Meeuwis et al., 2021.

Supplementary Table 3. Overview of mediation models: effects of verbal suggestions (VSs) on mean itch experienced during histamine iontophoresis, mediated by post-VSs itch expectation.

	n	path	Outcomes				
			M: post-VSs itch expectation [M(SE)]	path	Y: post-VSs mean itch [M(SE)]		
Study 1. Effects of open-label positive VSs versus neutral instructions							
constant	91		1.81 (0.48)***		2.43 (0.57)***		
X: group	91	a	-2.82 (0.29)***	c'	0.31 (0.43)		
M: itch expectation (post-VSs)	91			b	0.18 (0.09)*		
Cov1: expectation(pre-VSs)	91	e1	0.76 (0.08)***				
Model summary			$F(2,88) = 98.59$		$F(2,88) = 2.25$		
			$p < .001$		$p = .11$		
			$R^2 = .69$		$R^2 = .05$		
					95% bootstrapped CI		
					Lower Limit	Upper limit	
Indirect effect of X on Y	92	c	-0.51 (0.26)		-0.65	-0.03	
Study 2-3: open-label positive versus negative VSs							
constant	100		2.01 (0.52)***		0.33 (0.35)		
X: group	100	a	-1.72 (0.30)***	c'	-0.35 (0.26)		
M: itch expectation (post-VSs)	100			b	0.17 (0.08)*		
Cov1: itch expectation (pre-VSs)	100	e1	0.17 (0.09)†				
Cov2: baseline itch (pre-VSs)	100	e2	0.65 (0.09)***	e3	0.72 (0.09)***		
Model summary			$F(3,96) = 40.59$		$F(3,96) = 62.06$		
			$p < .001$		$p < .001$		
			$R^2 = .56$		$R^2 = .66$		
					95% bootstrapped CI		
					Lower Limit	Upper limit	
Indirect effect of X on Y	100	c	-0.29 (0.15)		-0.61	-0.02	
Study 2-3: closed-label positive versus negative VSs							
constant	103		1.29 (0.49)**		0.84 (0.36)*		
X: group	103	a	-1.84 (0.31)***	c'	-0.77 (0.31)*		
M: itch expectation (post-VSs)	103			b	0.011 (0.09)		
Cov1: itch expectation (pre-VSs)	103	e1	0.19 (0.08)*				
Cov2: baseline itch (pre-VSs)	103	e2	0.80 (0.08)***		0.82 (0.10)***		
Model summary			$F(3,99) = 41.53$		$F(3,99) = 42.29$		
			$p < .001$		$p < .001$		
			$R^2 = .56$		$R^2 = .56$		
					95% bootstrapped CI		
					Lower Limit	Upper limit	
Indirect effect of X on Y	103	c	-0.02 (0.19)		-0.46	0.30	

*Note. Percentile bootstrap CI based on 10,000 bootstrap samples. † $p < .10$; * $p < .05$; ** $p < .01$; *** $p < .001$*

References

Carver, C., and White, T.L. (1994). Behavioral inhibition, behavioral activation, and affective responses to impending reward and punishment: The BIS/BAS Scales. *The Journal of Personality and Social Psychology* 67, 319.

Cohen, S., Kamarck, T., and Mermelstein, R. (1983). A global measure of perceived stress. *Journal of Health and Social Behavior* 24, 385-396.

Eysenck, H.J., Eysenck, S.B.G. (1975). *EPQ (Eysenck Personality Questionnaire)*: Educational and Industrial Testing Service

Meeuwis, S.H., van Middendorp, H., Lavrijsen, A.P.M., Veldhuijzen, D.S., & Evers, A.W.M. (2021). Open-and closed-label placebo and nocebo suggestions about a sham transdermal patch. *Psychosomatic medicine* 83, 33.

Meeuwis, S.H., van Middendorp, H., van Laarhoven, A.I.M., Veldhuijzen, D.S., Lavrijsen, A.P.M., & Evers, A.W.M. (2019). Effects of open- and closed-label nocebo and placebo suggestions on itch and itch expectations. *Frontiers in Psychiatry* 10.

Meeuwis, S.H., van Middendorp, H., Veldhuijzen, D.S., van Laarhoven, A.I.M., De Houwer, J., Lavrijsen, A.P.M., & Evers, A.W.M. (2018). Placebo effects of open-label verbal suggestions on itch. *Acta dermato-venereologica* 98, 268-274.

Meyer, T.J., Miller, M.L., Metzger, R.L., and Borkovec, T. (1990). Development and validation of the penn state worry questionnaire. *Behaviour research and therapy* 28, 487-495.

Scheier, M., Carver, C., & Bridges, M. (1994). Distinguishing optimism from neuroticism (and trait anxiety, self-mastery, and self-esteem): a reevaluation of the Life Orientation Test. *Journal of personality and social psychology* 67, 1063-1078.

Van Beugen, S., Ograczyk, A., Ferwerda, M., Smit, J.V., Zeeuwen-Franssen, M.E., Kroft, E., De Jong, E.M., Zalewska-Janowska, A., T Donders, A.R., and Van De Kerkhof, P. (2015). Body attention, ignorance and awareness scale: assessing relevant concepts for physical and psychological functioning in psoriasis. *Acta dermato-venereologica* 95, 444-451

Zigmond, A.S., & Snaith, R.P. (1983). The Hospital Anxiety and Depression Scale. *Acta Psychiatrica Scandinavica* 67, 361-370.
